# Combined signaling of NF-kappaB and IL-17 contributes to Mesenchymal stem cells-mediated protection for Paraquat-induced acute lung injury

**DOI:** 10.1186/s12890-020-01232-5

**Published:** 2020-07-17

**Authors:** Lichun Zhang, Yu Wang, Haitao Shen, Min Zhao

**Affiliations:** Department of Emergency, Shengjing Affiliated Hospital of China Medical University, 36 Sanhao Street, Heping District, Shenyang, 110004 Liaoning Province China

**Keywords:** Paraquat (PQ), Oxidative stress, Mesenchymal stem cells (MSCs) transplantation, Acute lung injury (ALI), Pro-inflammatory response, Chronic pulmonary fibrosis

## Abstract

**Background:**

Paraquat (PQ) is an herbicide widely used in the world*.* PQ can cause pulmonary toxicity and even acute lung injury. Treatment for PQ poisoning in a timely manner is still a challenge for clinicians. Mesenchymal stem cell (MSC) transplantation has hold potentials for the treatment of several lung diseases including PQ poisoning. The aim of this study is to examine the mechanisms mediated by MSC transplantation to protect PQ-induced lung injury.

**Methods:**

Here we performed the whole genome sequencing and compared the genes and pathways in the lung that were altered by PQ or PQ together with MSC treatment.

**Results:**

The comparison in transcriptome identified a combined mitigation in NF-kappaB signaling and IL-17 signaling in MSC transplanted samples.

**Conclusion:**

This study not only reiterates the important role of NF-kappaB signaling and IL-17 signaling in the pathogenesis of PQ-induced toxicity, but also provides insight into a molecular basis of MSC administration for the treatment of PQ-induced toxicity.

## Background

Paraquat (1,1′-dimethyl-4,4′-bipyridinium dichloride, PQ) is a widely used herbicide in the world. PQ can cause fatal poisoning when absorbed via ingestion, skin contact, or inhalation. PQ poisoning has become a severe problem in some developing countries such as China [5]. Multiple organs such as liver, kidney, heart and central nervous system can be affected; but lung is the primary target of PQ [14, 20]. PQ is actively taken up in a concentration dependent manner and accumulates at particularly high levels in Clara cells and epithelial cells [5, 10, 13, 18]. PQ poisoning initially leads to acute lung injury (ALI), and ultimately to lung fibrosis or subsequent respiratory failure, which is the most common cause of death from PQ [12].

Paraquat undergoes redox cycling to generate hydrogen peroxide (H_2_O_2_) and highly toxic hydroxyl radicals (HO∙−) [23, 32]. These reactive oxygen species (ROS) induce stress responses and apoptosis via mitochondrial damage, lipid peroxidation and related signaling pathways such as Nrf2/ARE or NF-kBp65 pathway [1, 6]. Oxidative stress was considered one of potential mechanisms in which PQ induces ALI [3, 32]. Given the rapid effects of PQ and its highly reproducible animal model systems [25], PQ has been extensively used to study ALI and ALI-related diseases [30].

Previous studies indicated that mesenchymal stem cells (MSCs) are recruited to areas of lung injury, which further differentiate and repair pulmonary epithelial cells [21, 22]. MSCs can be derived from many sources, such as bone marrow, umbilical cord blood, dental pulp, adipose tissue and adult organs [2, 19]. These cells can be isolated via adherence to a plastic surface and share a common immuno-phenotype with CD105, CD73 and CD90 expression, but without CD45, CD34, CD14, CD19 and HLADR expression [2]. MSCs with low immunogenicity are used for xenogenous transplantation to achieve immunomodulation and improve tissue repair [19] [2]. Thus, MSC transplantation has also been a potential therapeutic approach for ALI induced by PQ poisoning [11]. MSC transplantation was showed to increase the survival time, decease the ratio of lung wet/dry weight and reduce the levels of inflammation and ROS production in rats upon PQ treatment [19]. Although MSC transplantation has been explored in a variety of pulmonary disease models, the molecular mechanisms that are beneficial for PQ-induced ALI have yet to be understood [28].

Recently, gene expression study reveals insight into the molecular mechanism of PQ-induced toxicity [19]. To identify transcriptional changes and pathway alterations that can potentially protect PQ-induced lung toxicity by MSC transplantation, we analyzed and compared the gene expression profiles between PQ-induced ALI models with and without MSC transplantation. The analyses identified NF-kappaB signaling and IL-17 signaling that were selectively altered by MSC transplantation upon treatment of PQ. Thus, our study demonstrated the potential genes or signaling pathways that might function to protect PQ-induced ALI by MSC transplantation, which provides an explanation for the beneficial effects of MSC transplantation on PQ-induced ALI.

## Methods

### Animals and treatment

Female Sprague-Dawley (SD) rats (3 males, 120 g + 20) in each group were provided by the Experiment Animal Center of the Shengjing Affiliated Hospital of China Medical University (Shengyang, China) and our animal research was conducted according to ethical guidelines. Housing, husbandry conditins and welfare-related assessments and intervention were followed by experimental animal protocols of the Shengjing Affiliated Hospital of China Medical University.200 g/l PQ dichloride solution was obtained from Shengyang Agriculture Pesticides Technology & Development Inc. (Shengyang, China). Rats were euthanized by intraperitoneal injection of sodium pentobarbital at a dose of > 100 mg/kg body weight 7 days after PQ injection according to experimental animal protocols of the Shengjing Affiliated Hospital of China Medical University. The study was approved by Ethics Review Committee of the Shengjing Affiliated Hospital of China Medical University.

GFP expressing Bone marrow MSCs were purchased from Cyagen (Bejing, China). The surface expressions of CD34, CD45, CD44 and CD166 on MSC cells were measured by flow cytometry. The SD rats (recipients) were randomly divided into three groups: (1) normal control group, which were given an equivalent volume of 0.9% physiological saline by intraperitoneal injection; (2) PQ group, which were given 200 g/l PQ at 15 mg/kg; (3) MSC and PQ treated group, which were given MSC (10 million) by intravenous injection at 6 h before PQ exposure. Injected MSCs were followed by fluorescent microscope in lung tissues (Olympus FV-1000).

### Pathology

The right lung lobes were removed from experimental animals at different times after they were injected with PQ. Tissues were fixed with 10% neutral-buffered formalin through intracheal perfusion and embedded in paraffin. Sections of 5 μm thickness were subjected to H&E staining.

### RNA extraction and quality control

Total RNA from lung tissues (3 animals from each experimental group) was isolated using TruSeq Stranded Total RNA Library Prep Kit (Illumina, CA, USA) and TruSeq small RNA sample Preparation kit (Illumina, CA, USA) according to the manufacturer’s instructions. RNA quantification, quality and integrity were checked with Qubit, NanoDrop and Agilent 2100 Bioanalyzer (Agilent Technologies, Santa Clara, CA, USA) according to the manufacturer’s instructions.

### Gene expression profiling

Gene expression profiling was performed with service from Genesky Biotech (Shanghai, China). Briefly, total RNA was used to deplete rRNA, purify and fragmentation RNA. Sequence libraries are then prepared by ligating specialized adapters to both ends of fragmented cDNAs, which were reversely transcribed from RNAs. The libraries were then subjected to sequencing using Illumina HiSeq 3000 (Illumina, CA, USA) with paired-end reads. Raw data reads were evaluated by Software FastQC to obtain Q value to represent quality of the reads. Reads were then filtered using TrimGalore to remove sequences of primers, sequences of fragments less than 35 bp and sequences with quality Q less than 10. Totally reads or fragments were mapped for an alignment with known Sprague-Dawley rat sequence using software HISAT2 (*https://ccb.jhu.edu/software/hisat2/manual.shtml**)*. Different gene expression levels among different samples were calculated by Cufflinks (http://cole-trapnelllab.github.io/cufflinks/referred).

### Statistical analysis of gene expression profile

Different expression levels of the samples were performed using one-way analysis of variance (ANOVA). *p* values were adjusted for multiple comparisons using false discovery rate multiple testing correction. Differentially expressed genes were selected with threshold of relative ± twofold change and adjusted *p* values ≤0.05.

The heat map was generated using Heatmap.2 available in “gplots” package in R program. Function and pathway analysis of differentially expressed genes were analyzed with canonical pathways and gene ontology (GO) biological processes associated with identified differentially expressed genes, which were disclosed using MetaCore GeneGO server (https://portal.genego.com/). p values were calculated based on hypergeometric distribution and reflected the probability for a pathway or process to arise by chance. Pathways and processes with a Benjamini-Hochberg multiple testing correction *p* value of ≤ 0.05 were considered significant.

## Results

### MSC characteristics

**Fig. 1:**
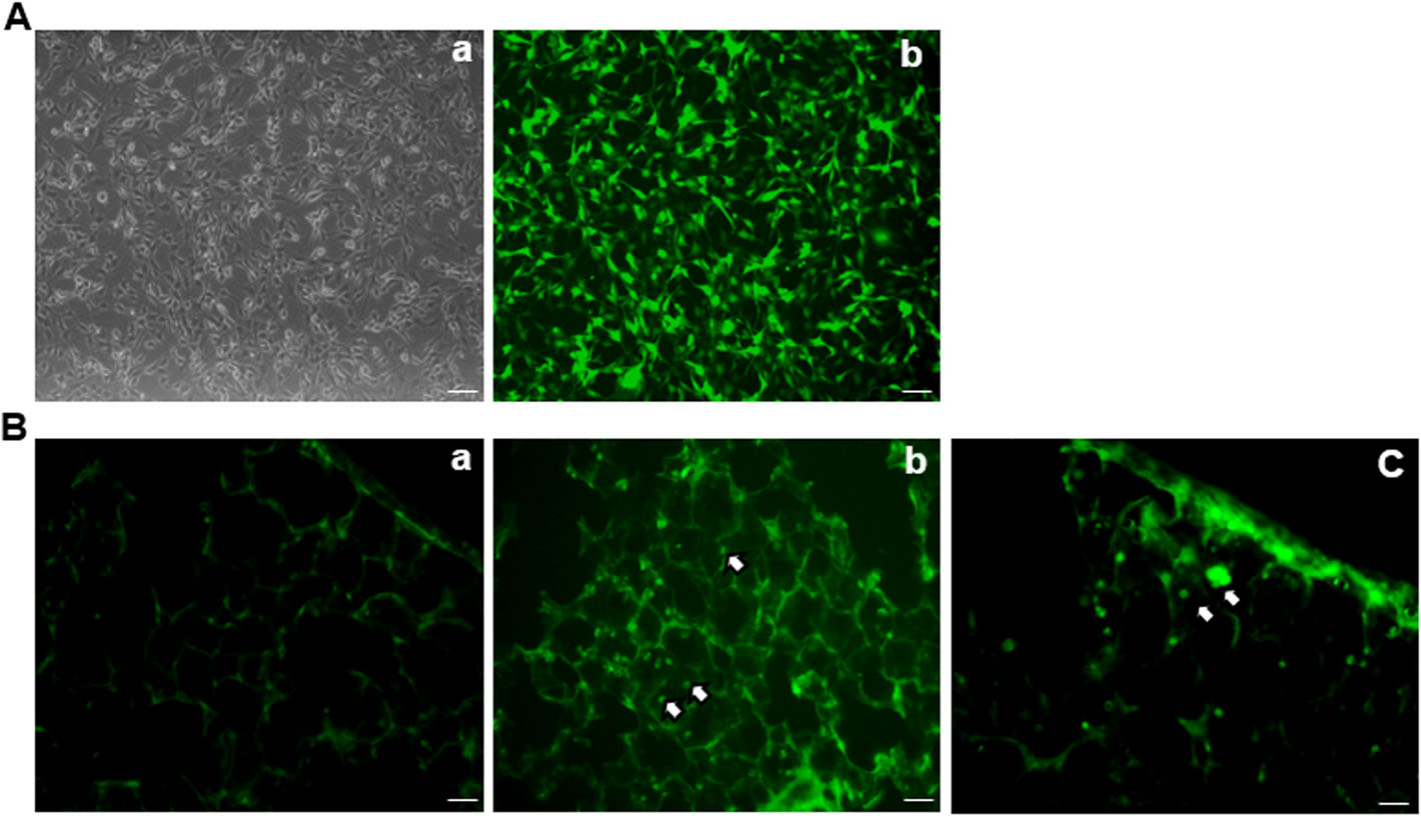
Mesenchymal stem cells (MSCs) were transplanted into paraquat (PQ)-treated rats. **a** Representative images of MSCs were observed under bright field (**a**) or fluorescent (**b**) microscope, scale bar: 50 μm; **b** Representative fluorescent images of lung section from control group (**a**) or MSC transplanted group (b&c)

MSCs were known to improve the survival rates of animals with treatment of PQ. The characteristics of MSCs are important for their beneficial effects on PQ-poisoned animals. The qualification report of the batch MSCs in this study was included in Table S1. MSCs with green florescent protein (GFP) overexpression were used in this study to monitor the efficiency of the MSC incorporation into the lung after the injection. Florescent signals were detected in animals with MSC injection (Fig. 1), indicating that MSCs can reach lung tissues and protect them from the toxic effects of PQ.

### MSCs alleviate ALI induced by PQ

**Fig. 2:**
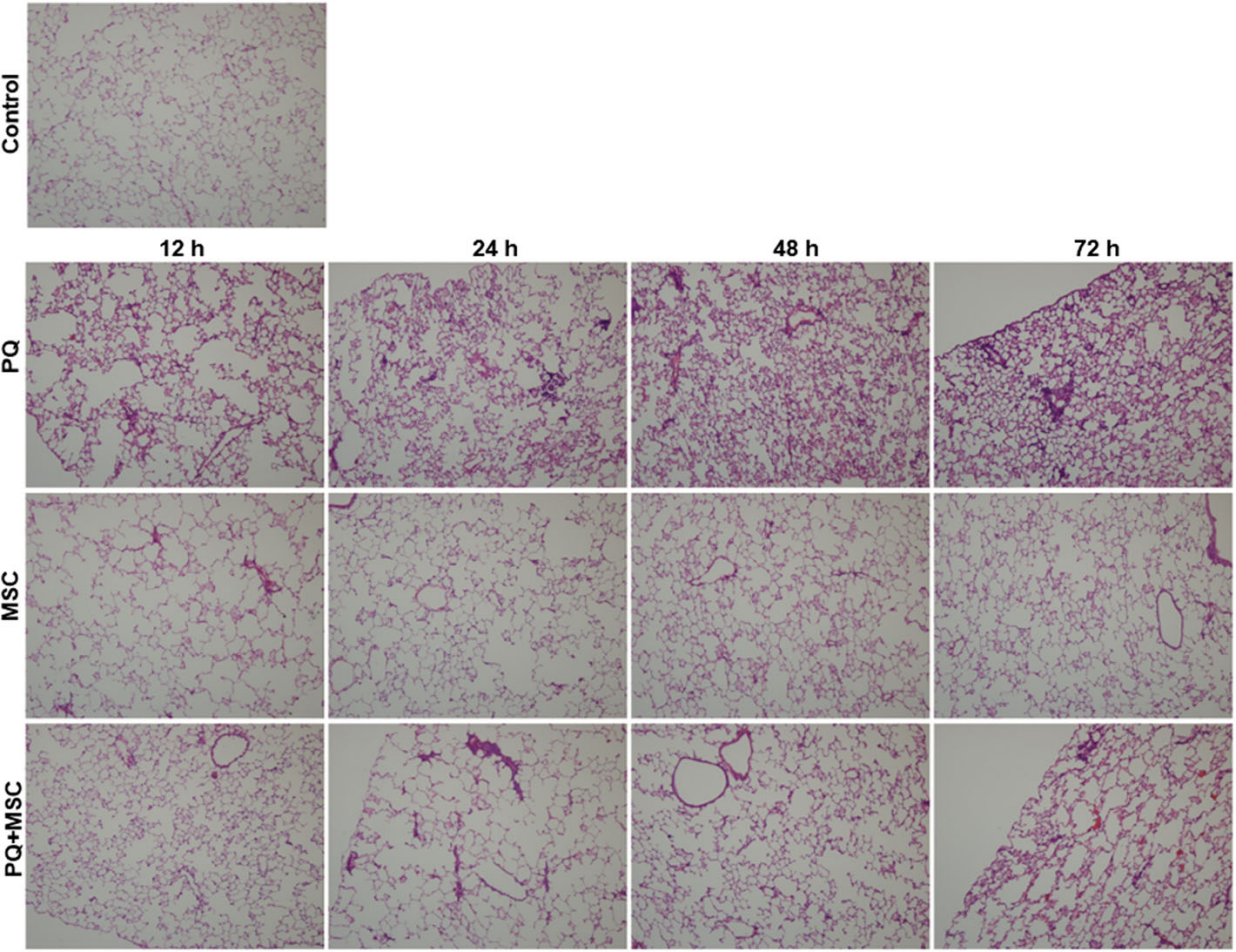
MSC transplantation protects paraquat (PQ)-induced lung injury. H&E staining was performed on lung sections from different group of rats: Control for untreated rats; PQ for paraquat-treated rats; MSC for MSC-transplanted rats; PQ + MSC for MSC-transplanted paraquat-treated rats

The development of induced lung damages includes an acute-phase response immediately after PQ treatment and a followed progression to chronic fibrosis. Our H&E staining results indeed showed an increased damage over the time course of PQ treatment and peaked at 7 day (Fig. 2). Pre-treatment of MSCs significantly alleviated the toxic effects of PQ treatment on damages (Fig. 2), which is consistent with the previously published reports.

### Transcriptome changes in response to PQ treatment

**Fig. 3:**
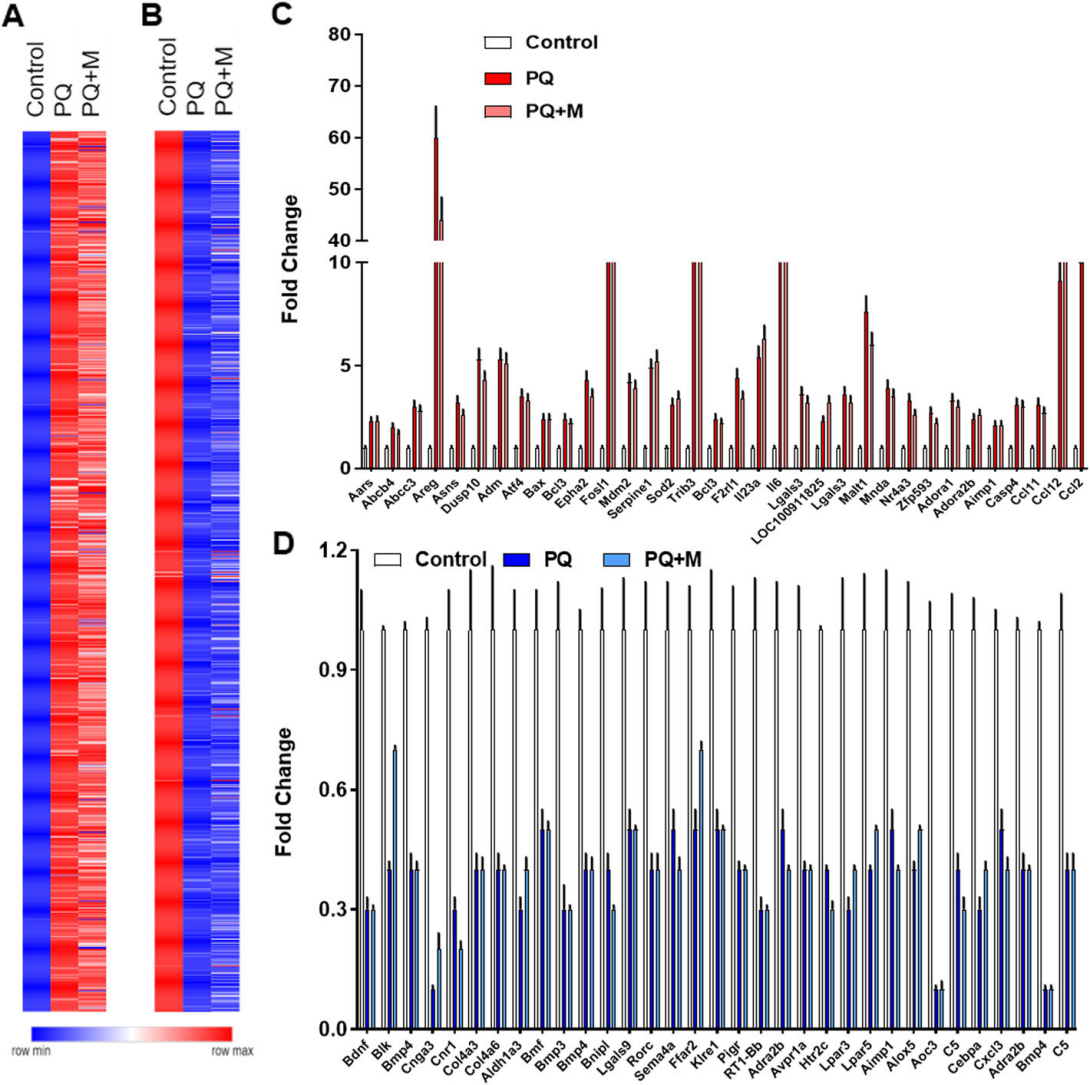
Gene expression profiles among different paraquat-treated groups. **a** and **b** Heat maps were generated for the up-regulated genes (**a**) and down-regulated genes (**b**) in paraquat (PQ)-treated rats with MSC transplantation (PQ + MSC) or without (PQ); Commonly up-regulated genes in PQ and PQ + MSC groups were selectively plots (mean ± SD, *n* = 3) in **c**) and commonly down-regulated genes in PQ and PQ + MSC groups were selectively plots (mean ± SD, n = 3) in **d**)

After the treatment with PQ for 7 days, the acute responses start to transit to fibrogenesis, which can be protected by MSC injection. To identify the critical genes or pathways that mediate the protection by MSCs for PQ-induced toxicity, we performed the whole genome sequencing and compared the genes or pathways in lungs that were altered by PQ or PQ together with pre-injection of MSCs. 1-1Treatment with PQ affected the expression of 1797 genes, in which 916 were up-regulated and 881 down-regulated as compared with the saline control. Pre-injection of MSCs slightly decreased the numbers of genes affected with 809 genes up-regulated and 831 genes down-regulated. Relative gene expression levels were compared by the Heat Map analysis showed in Fig. 3a, and detailed gene expression levels were listed in Table S2. According to the Heat Map, the changes in gene expression patterns are largely similar between PQ-treated samples with or without pre-injection of MSCs, although the extent of changes were noticeably reduced among a portion of genes in samples with pre-injection of MSCs (Fig. 3a). Fold changes in expression levels were exemplified for a group of genes from different pathways (Aars, Abcb4, Abcc3, Areg, Asns, Dusp10, Bdnf, Blk, Bmp4, Cnga3, Cnr1, Col4a3 and Col4a6 for response to oxidative stress; Adm, Atf4, Bax, Bcl3, Epha2, Fosl1, Mdm2, Serpine1, Sod2, Trib3, Bmf, Aldh1a3, Bmp3, Bmp4, Bnipl for apoptotic process; Bcl3, F2rl1, F2rl1, Il23a, Il6, Lgals3 Lgals9, Rorc and Sema4a for T-cell activation involved in immune response; LOC100911825, Lgals3, Malt1, Mnda, Nr4a3, Zfp593, Adora1, Adora2b, Aimp1, Casp4, Ccl11, Ccl12, Ccl2 RT1-Bb, Adra2b, Avpr1a, Htr2c, Lpar3, Lpar5, Aimp1, Alox5, Aoc3, C5, Cebpa, Cxcl3, Adra2b, Bmp4, C5 and Cadm1 for inflammatory response) in Fig. 3b.

### GO and KEGG pathway analysis

**Fig. 4:**
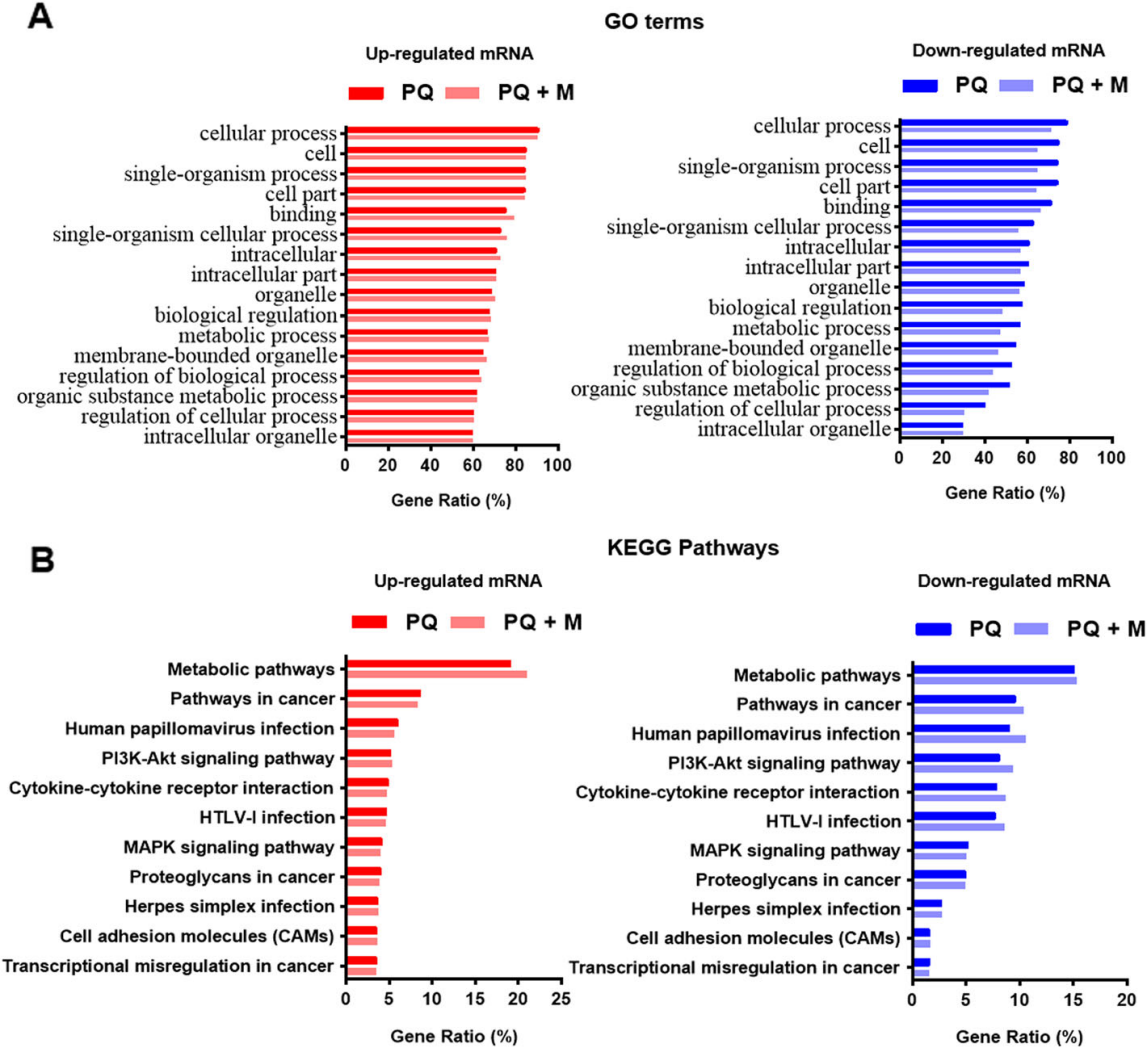
GO and KEGG pathway analysis. **a** The top GO terms for RNAs that are most enriched in up-regulated (right) or down-regulated (left) in paraquat (PQ)-treated rats with MSC transplantation (PQ + MSC) or without (PQ); **b** The top KEGG terms for RNAs that are most enriched in up-regulated (right) or down-regulated (left) in paraquat (PQ)-treated rats with MSC transplantation (PQ + MSC) or without (PQ)

To obtain more insights into how those up- or down- regulated genes may contribute to the pathogenesis of paraquat-induced toxicity, we first performed functional analysis of the gene profiling results. Gene Ontology analysis revealed that the statistically significantly enriched functional terms include a variety of pathways involved in different stress responses and immune responses such as oxidative stress responses, drug substance responses, T-cell mediated immune responses, T-cell activation or differentiation mediated immune response and even innate immune response. Those stress responses are carried by a series of signal transduction cascades including G-protein coupled receptor pathways, integrin-mediated signaling pathway, I-kappaB kinase/NF-kappaB signaling, JAK-STAT cascade, Wnt signaling pathway, DNA damage response, signal transduction by p53 class mediator, toll-like receptor 4 signaling pathway, p38MAPK cascade, ERK1 and ERK2 cascade, regulation of ERBB signaling pathways according to KEGG pathway analysis. Top enrichment of GO terms and KEGG pathways for both up- or down- regulated genes were summarized in Fig. 4a and Fig. 4b. More than 8 thousand GO terms and 80 KEGG pathways were found among up- or down- regulated genes. Detailed information was included in Table S3 and Table S4. These observations demonstrate that PQ treatment induces significant changes in expression of genes involved in numerous signaling transduction pathways of oxidative stress and inflammation in the lungs. The pathways that were dramatically changed in the upregulated or downregulated mode were shared, reinforcing the importance of those pathways in the response to PQ.

Changes in lung gene expression by pre-injection of MSCs upon PQ treatment.

**Fig. 5:**
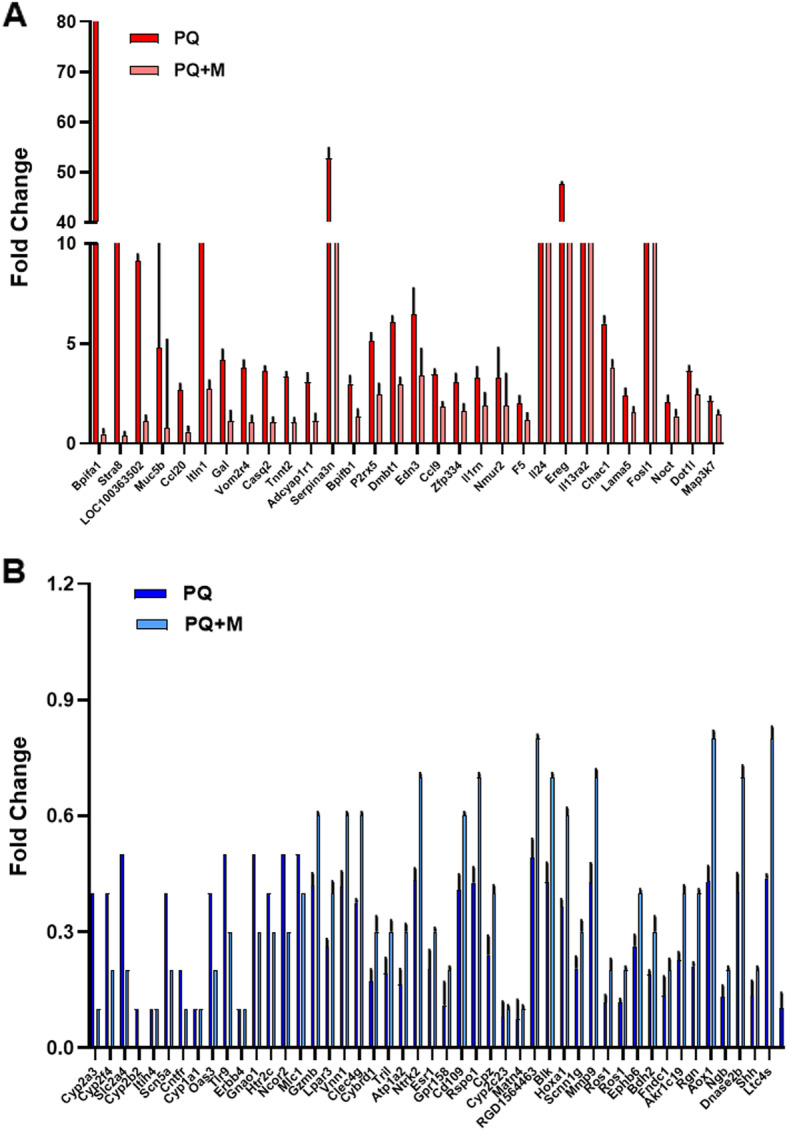
Different gene expression between PQ-treated samples with and without transplantation of MSCs. Up- (**a**) or down- (**b**) regulated genes that were increased or decreased to less extent in samples with transplantation of MSCs were selectively plotted (mean ± SD, n = 3)

To further identify the mechanisms that could protect from PQ treatment by MSCs, we performed quantitative comparisons of regulated genes between samples with and without pre-injection of MSCs. Surprisingly, only 111 genes were differentially expressed between PQ-treated samples with or without pre-injection of MSCs. Figure 5a and Fig. 5b demonstrate either up- or down- regulated genes that were increased or decreased to less extent in samples with pre-injection of MSCs. These up-regulated genes mainly include Bpifa1, Stra8, LOC100363502, Muc5b, Ccl20, Itln1, Gal, Vom2r4, Casq2, Tnnt2, Adcyap1r1, Serpina3n, Bpifb1, P2rx5, Dmbt1, Edn3, Ccl9, Zfp334, Il1rn, Nmur2, F5, Il24, Ereg, Il13ra2, Chac1, Lama5, Fosl1, Noct, Dot1l, Map 3 k7. These down-regulated genes mainly include Cyp2a3, Cyp2f4, Slc2a4, Cyp2b2, Itih4.

**Fig. 6:**
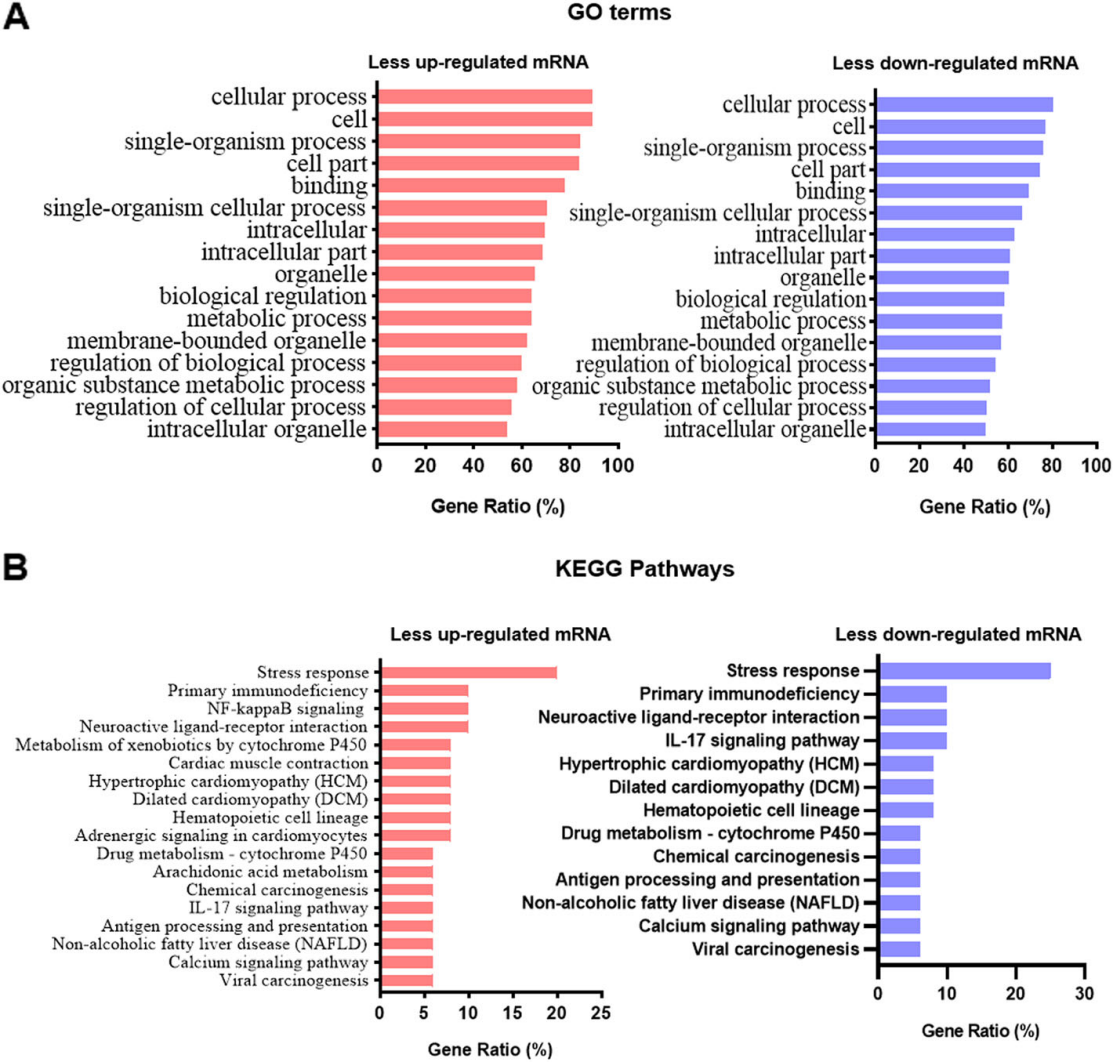
GO and KEGG pathway analysis for different gene expression between PQ-treated samples with and without transplantation of MSCs. **a** The top GO terms for differently expressed RNAs that are most enriched in less up-regulated (right) or down-regulated (left) in MSC-transplanted paraquat (PQ)-treated rats; **b** The top KEGG terms for different expressed RNAs that are most enriched in less up-regulated (right) or down-regulated (left) in MSC-transplanted paraquat (PQ)-treated rats

Scn5a, Cntfr, Cyp1a1, Oas3, Tlr9, Erbb4, Gnao1, Htr2c, Ncor2, Mlc1 Gzmb, Lpar3, Vnn1, Clec4g, Cybrd1, Tril, Atp1a2, Ntrk2, Esr1, Gpr158, Cd109, Rspo1, Cpz, Cyp2c23, Matn4, RGD1564463, Blk, Hoxa1, Scnn1g, Mmp9, Ros1, Ephb6, Bdh2, Fndc1, Akr1c19, Rgn, Aox1, Ngb, Dnase2b, Shh, Ltc4s, Hsd11b2. Gene Ontology and KEGG pathway analysis showed that those differently expressed genes are highly enriched in oxidative stress responses such as Bpifa1, Stra8, Itln1, Gal, Casq2, Tnnt2, Adcyap1r1, Serpina3n, Gnao1, Hspg2, Cyp2e1, Cnga3 and immune responses such as Bpifb1, Ccl9, Il1rn, Il24, Il13ra2, Tlr9, Gzmb, Vnn1, Tril, Ntrk2, Esr1, Blk, Tnfrsf13c. Among those pathways, NIK/NF-kappaB signaling and IL-17 signaling pathway appear to be critically changed in the gene expression between PQ-treated samples with and without pre-injection of MSCs in Fig. 6a and Fig. 6b. This observation indicated an important role of NF-kappaB and IL-17 signaling in the pathogenesis of PQ-induced acute lung injury. It is likely that MSCs protect from paraquat-induced toxicity through ameliorating both NF-kappaB signaling and IL-17 signaling.

Mitigated NIK/NF-kappaB signaling and IL-17 signaling pathways in MSC transplanted samples.

**Fig. 7:**
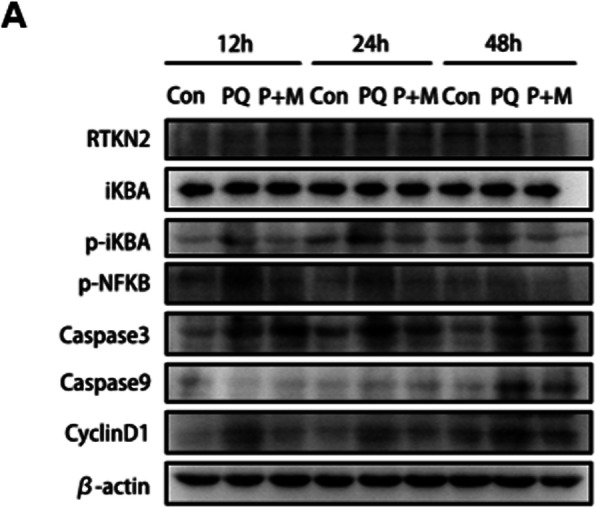
Mitigated NIK/NF-kappaB signaling and IL-17 signaling pathways in MSC transplanted samples. Protein samples were analyzed by Western blotting against antibodies of RTKN2, total and phosphorylation of IκBα, phosphorylation of NF-kappaB, Caspase3, Caspase9, CyclinD1 and β-actin. Protein samples were extracted from lungs of different experimental groups treated with PQ (Con: Control group; PQ: paraquat-treated group; P + M: PQ treatment with MSC transplantation group) at different time points, 12 h, 24 h and 48 h

To further confirm the protective role of NIK/NF-kappaB and IL-17 pathways in MSC transplanted samples, we analyzed key signaling events in the pathways such as phosphorylation of IκBα, phosphorylation of NF-kappaB and upregulation of caspase3, caspase9 and CyclinD1 at protein levels by Western blotting. Phosphorylation of IκBα and phosphorylation of NF-kappaB were significantly increased upon PQ treatment at different time points tested, 12 h, 24 h and 48 h, which is attenuated in the MSC transplanted samples, especially at 12 h and 24 h (Fig. 7 and Fig.S1). Protein levels of caspase3, caspase9, and CyclinD1 were also induced over the time course of PQ treatment, which was reduced in the MSC transplanted samples. This observation indicates mitigated NIK/NF-kappaB and IL-17 signaling pathways in MSC transplanted samples upon PQ-treatment. These results suggest that transcriptional changes identified by the whole genome sequencing are reflected in the protein and signaling pathways.

Changes in non-coding RNA expression by PQ-treated samples.

Non-coding RNA (ncRNAs) are RNA molecules without translated protein products. There is.

limited study on the expression profile of those non-coding RNAs in PQ-treated samples. In this set of gene profiling experiments, we also included the reports on those RNA levels (Table S5). Unfortunately, we were not able to identify any significant difference in their levels between PQ-treated samples with and without pre-injection of MSCs. This observation suggests that those non-coding RNAs may not significantly contribute to the regulation of protection mediated by MSCs for PQ-induced toxicity.

## Discussion

Both biological research and clinical studies have shown that the lung is the primary organ for PQ-induced toxicity. Pulmonary cells can actively intake and accumulate PQ [17] [4]. The acute lung injury and the followed fibrosis are the main causes for PQ-induced toxicity [11] [15]. Treatment of the acute lung injury can effectively prevent the fibrosis afterwards [15]. Recent treatments include: (1) removing the poison from the gastrointestinal tract or reducing absorption through gastric lavage, catharsis, or oral administration; (2) blood purification to remove PQ; (3) immunosuppressants therapy with high-dose glucocorticosteroids; (4) removal of oxygen radicals, produced by PQ-induced damage; (5) lung transplantation. Although patient survival has been improved by those therapies since 1987, mortality is still a concern for clinicians [7, 15, 17]. Therefore, the mechanistic study of PQ poisoning and new therapeutic strategies may help further develop treatment strategies.

As stem cell therapies have been applied in different disease areas, stem cell-related treatment has also drawn a wild attention in lung injury field. MSCs are excellent source for seeding to initiate pulmonary cells to proliferate and differentiate [19]. MSCs are easily acquired and readily to be administered with a strong regeneration capacity, low levels of caducity and immune-mediated rejection [28]. Previously, numerous studies indicated that MSCs can be recruited to areas of lung injury, where they differentiate and repair pulmonary epithelial cells, increase the secretion of surface-active substances, and reduce inflammatory reactions [29]. Moreover, MSC transplantation was shown to increase the survival time of PQ-treated rats, decease the ratio of lung wet/dry weight, reduce the levels of TNF-a, IL-1 b and MDA [26], and reduce the consumption of SOD and GSH-PX (particularly by day 7 post-transplantation) [2]. MSC transplantation can physically reduce lung edema, inhibit the release of inflammatory cytokines and suppress inflammatory responses. Therefore, MSCs have proved the feasibility to protect from PQ-induced lung injury. However, the protective mechanisms that are mediated by MSC transplantation are still largely unknown [11] [6].

Oxidative stress is considered as one of the major mechanisms underlying PQ poisoning. PQ not only can alter redox potentials and induce intensive oxidative stress responses [6], but also can directly oxidize IkB and degrade the IkB-NF-kB complex to activate NF-kB, which further initiate and escalate the crosstalk between oxidative stress and inflammatory responses with upregulation of TNFα and IL-1b [1]. Activation of those signaling pathways was exactly observed in our gene profiling experiments. NF-kB signaling is a signaling node that initiates pulmonary inflammation to an extent that leads to a detrimental toxicity for the acute lung injury [8, 9, 24]. Interestingly, NF-kB signaling was significantly attenuated in MSC transplanted samples according to our whole transcriptomic analysis, which was further conformed by Western blotting analysis. This observation confirms the causal effects of NF-kB signaling in the pathogenesis of PQ-induced toxicity [23], which is consistent with previous results [23]. This observation also suggests that MSC transplantation can efficiently mitigate the initial signaling events for the further exacerbation of the toxic effects of PQ.

Interleukin 17A (IL-17A) is a pro-inflammatory cytokine that regulates the host defense against several different pathogens [25, 27]. IL-17A plays an important role in the recruitment of neutrophils and other immune cells to the infection sites. Different cells are known to produce IL-17A including NK cells, γδ T cells, T helper (Th)17, innate immune cells and even neutrophils to participate different immune responses. Recent studies showed that T cells are involved in a variety of aseptic inflammation and autoimmune diseases in an IL17A-dependent manner. In addition, it was reported that HMGB1-TLR4-IL23-IL17A axis promotes paraquat-induced ALI by mediating neutrophil infiltration in mice [27]. Therefore, IL-17A signaling pathways are essential to propagate the immune responses triggered by PQ treatment [25]. Consistent with the role of IL-17A in the pathogenesis, our transcriptomic results showed a dramatic down-regulation of genes in IL-17A signaling pathways from MSCs transplanted samples [31]. This observation confirms the importance of IL-17A in the pathogenesis of PQ-induced toxicity [31]. In addition, this observation pinpoints the protective role of MSCs in PQ toxicity through tempering the immune response mediated by IL-17A [16].

A series of systematic preclinical studies showed that transplanted MSCs can modulate inflammatory cytokines in the blood or local lung tissues via paracrine secretion functions and thus play anti-inflammatory and anti-fibrosis roles in the lung. Although there are clinical challenges to apply MSC transplantation to the patients such as adequate time for MSC preparation and the implementation of treatment policy, MSCs could still be a new and effective biological agent for the treatment of clinical PQ poisoning. Our study provides the molecular fundamentals from whole transcriptome scope for further efforts in MSC transplantation research and clinical trials in PQ treatment.

## Conclusion

PQ is a widely used herbicide in the world, which can cause pulmonary toxicity and acute lung injury. Treatment for PQ poisoning is still a challenge for clinicians, especially in China rural areas. Stem cell transplantation has been tested for the treatment of several lung diseases including PQ poisoning and therapeutic effects were observed. Through the transcriptomic analysis, this study probed MSC-mediated mechanisms in the role of protecting from PQ poisoning. The comparison in transcriptome and molecular events identified a combined mitigation in NF-kappaB signaling and IL-17 signaling in MSC transplanted samples. This study pinpoints an important role of NF-kappaB signaling and IL-17 signaling in the pathogenesis of PQ-induced toxicity, which provides an explanation on molecular basis of MSC-mediated treatment for PQ poisoning.

## Supplementary information

**Additional file 1.**

**Additional file 2.**

**Additional file 3.**

**Additional file 4.**

**Additional file 5.**

**Additional file 6.**

**Additional file 7.**

## Data Availability

Data will be available upon the request.

## Abbreviations

PQ: Paraquat
MSCs: Mesenchymal Stem Cells
ALI: Acute Lung Injury
ROS: Reactive oxygen species
IL-17A: Interleukin 17A
GO: Gene ontology
