# Supplementary figures and images for: Combined signaling of NF-kappaB and IL-17 contributes to Mesenchymal stem cells-mediated protection for Paraquat-induced acute lung injury
Source: BMC Pulm Med. 2020 Jul 17;20:195. doi: 10.1186/s12890-020-01232-5 (PMC7367411; doi:10.1186/s12890-020-01232-5)

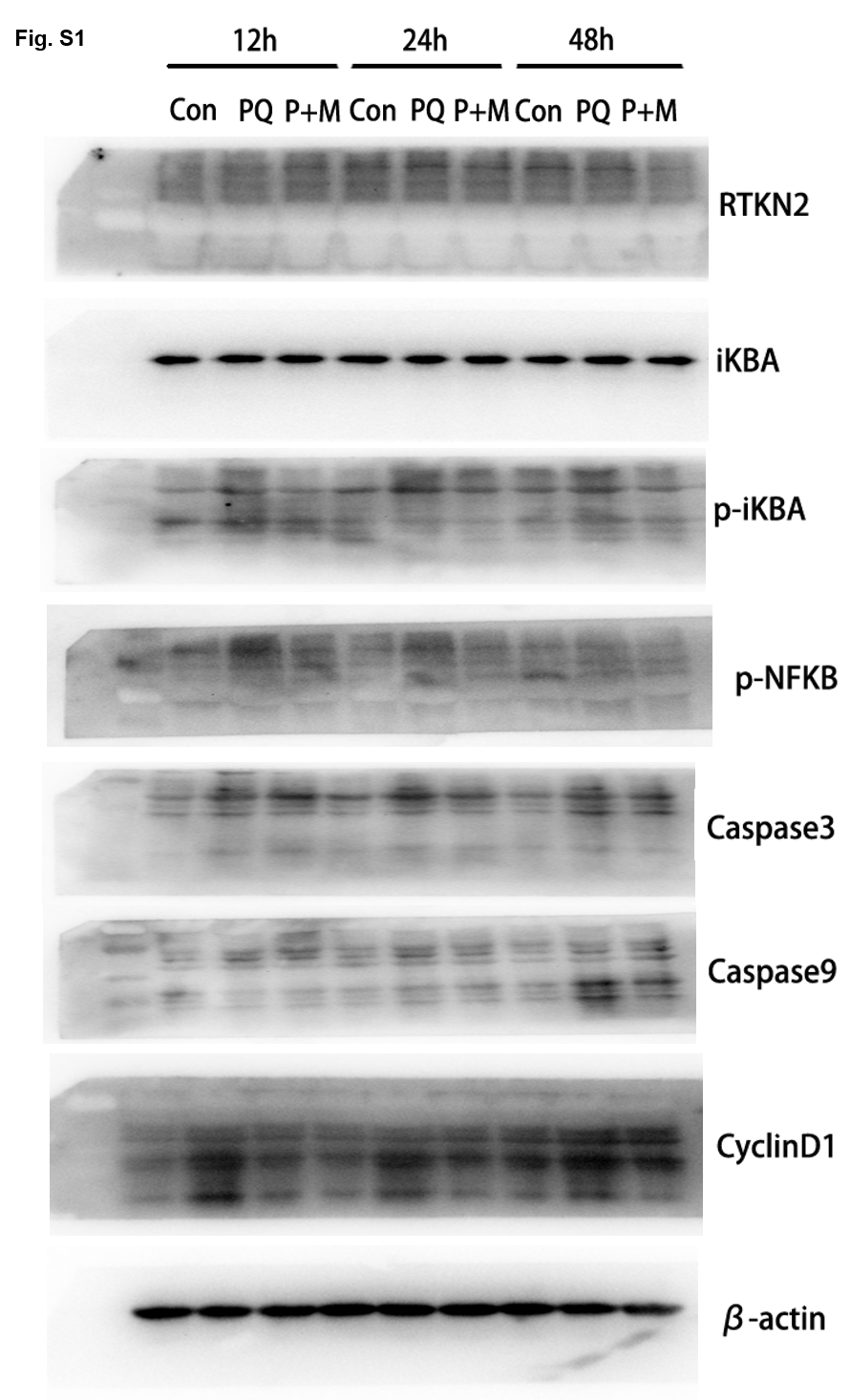

Supplement: Supplementary file 6 — Additional file 6. [file 12890_2020_1232_MOESM6_ESM.tif]
