# Supplementary material for: Combined signaling of NF-kappaB and IL-17 contributes to Mesenchymal stem cells-mediated protection for Paraquat-induced acute lung injury
Source: BMC Pulm Med. 2020 Jul 17;20:195. doi: 10.1186/s12890-020-01232-5 (PMC7367411; doi:10.1186/s12890-020-01232-5)
Supplement: Supplementary file 7 — Additional file 7. [file 12890_2020_1232_MOESM7_ESM.docx]

**Combined Signaling of NF-kappaB and IL-17**

**Contributes to Mesenchymal Stem Cells-Mediated Protection for**

**Paraquat-induced Acute Lung Injury**

Lichun Zhang^1^, Yu Wang^1^, Haitao Shen^1^, Min Zhao^1^*

^1^Department of Emergency, Shengjing Affiliated Hospital of China Medical University

36 Sanhao Street, Heping District, Shenyang, Liaoning Province 110004, China

Tel: +8624-96615-64112; 18940251586

Fax number：+8624-96615-64131

Lichun Zhang: [lichunzhangcmu@163.com](mailto:lichunzhangcmu@163.com)

Yu Wang, MD: [wangy8@sj-hospital.org](mailto:wangy8@sj-hospital.org)

Haitao Shen, MD: [shenht@sj-hospital.org](mailto:shenht@sj-hospital.org)

Min Zhao, MD: [zhaom@sj-hospital.org](mailto:zhaom@sj-hospital.org)

**Key Word**: **Paraquat (PQ),** **Oxidative Stress, Mesenchymal Stem Cells (MSCs) Transplantation, Acute Lung Injury (ALI), Pro-inflammatory Response, Chronic Pulmonary Fibrosis**

**[*Corresponding author: zhaomsy@163.com](mailto:*Corresponding author: zhaomsy@163.com)**

**Supplemental Materials**

**Fig. S1 Mitigated NIK/NF-kappaB signaling and IL-17 signaling pathways in MSC transplanted samples**

Protein samples were analyzed by Western blotting against antibodies of RTKN2, total and phosphorylation of IκBα, phosphorylation of NF-kappaB, Caspase3, Caspase9, CyclinD1 and β-actin. Protein samples were extracted from lungs of different experimental groups treated with PQ (Con: Control group; PQ: paraquat-treated group; P+M: PQ treatment with MSC transplantation group) at different time points, 12 h, 24 h and 48 h.
